# Unbiased RNA-Seq-driven identification and validation of reference genes for quantitative RT-PCR analyses of pooled cancer exosomes

**DOI:** 10.1186/s12864-020-07318-y

**Published:** 2021-01-06

**Authors:** Yao Dai, Yumeng Cao, Jens Köhler, Aiping Lu, Shaohua Xu, Haiyun Wang

**Affiliations:** 1 Department of Gynecology, Shanghai First Maternity and Infant Hospital, School of Life Sciences and Technology, Tongji University, Shanghai, 200092 China; 2 Department of Medical Oncology, Dana-Farber Cancer Institute, Boston, MA 02215 USA

**Keywords:** Reference gene, qRT-PCR, Cancer exosome, RNA-Seq, miRNA-Seq

## Abstract

**Background:**

Exosomes are extracellular vesicles (EVs) derived from endocytic compartments of eukaryotic cells which contain various biomolecules like mRNAs or miRNAs. Exosomes influence the biologic behaviour and progression of malignancies and are promising candidates as non-invasive diagnostic biomarkers or as targets for therapeutic interventions. Usually, quantitative real-time polymerase chain reaction (qRT-PCR) is used to assess gene expression in cancer exosomes, however, the ideal reference genes for normalization yet remain to be identified.

**Results:**

In this study, we performed an unbiased analysis of high-throughput mRNA and miRNA-sequencing data from exosomes of patients with various cancer types and identify candidate reference genes and miRNAs in cancer exosomes. The expression stability of these candidate reference genes was evaluated by the coefficient of variation “CV” and the average expression stability value “M”. We subsequently validated these candidate reference genes in exosomes from an independent cohort of ovarian cancer patients and healthy control individuals by qRT-PCR.

**Conclusions:**

Our study identifies *OAZ1* and *hsa-miR-6835-3p* as the most reliable individual reference genes for mRNA and miRNA quantification, respectively. For superior accuracy, we recommend the use of a combination of reference genes - *OAZ1*/*SERF2*/*MPP1* for mRNA and *hsa-miR-6835-3p*/*hsa-miR-4468-3p* for miRNA analyses.

## Background

Exosomes are a class of extracellular vesicles (EVs) which are secreted by eukaryotic cells. Exosomes contain biomolecules, such as DNA, RNA, miRNA or proteins and are considered important mediators of intercellular communication [1–7]. Cancer cell-derived exosomes play a pivotal role in tumorigenesis and cancer progression as they modulate cancer cell biology, the tumor microenvironment and the immune response [7–13]. Tumor-derived exosomes can also be harnessed as non-invasive diagnostic biomarkers due to their abundance in biological fluids and the enrichment of tumor-relevant biomolecules such as mRNAs or miRNAs within [4, 14–17]. In the past, various exosome-based liquid biopsies studies have suggested clinical feasibility for cancer diagnosis [18–20].

To accurately explore exosomes as non-invasive biomarkers and to better understand their impact on cancer progression, the precise quantification of biomolecule abundance within exosomes is of utmost importance. Quantitative real-time polymerase chain reaction (qRT-PCR) is the most widely used laboratory technique to evaluate cell-intrinsic and exosomal gene expression patterns [21]. qRT-PCR offers the advantage of high sensitivity and specificity combined with reproducibility and low template input requirements [22, 23]. However, technical or experimental factors inherent to qRT-PCR, such as variable template integrity or efficiency of reverse transcription, can reduce the diagnostic accuracy [23–26]. In addition, the numbers, sizes, and compositions of exosomes are usually affected by many factors including the methodologies for exosome isolation, intracellular biological processes, cell culture parameters and the treatments of the parental cells, which introduce the difficulty for the characterization of the composition in exosomes [27–29]. To account for this, reference genes with stable expression across different conditions or cancer subtypes are used to normalize gene expression [22, 30, 31]. Currently, the reference genes used for expression analyses in exosomes are most frequently those which are also used for tissue or cell lines, such as *ACTB*, *18S rRNA* and *GAPDH* [5, 32, 33]. Notwithstanding their broad use, expression levels of these housekeeping genes are not universally stable, thus decreasing the quantitative accuracy in exosome studies [22, 31, 34–37]. For example, the small nucleolar RNA *RNU6* is frequently used as a reference gene for miRNA expression analyses within cells [38–40], but the molecule is only expressed in the cell nucleus and not detected in exosomes [41–43]. Whereas some studies reported *RNU6* to be detectable in exosomes, this is most likely due to contamination of the exosome fraction with intact cells or cell debris [44, 45]. Therefore, the unvalidated use of classical housekeeping genes suitable for cell lines or tissues needs to be critically considered for the analysis of exosomes.

To address this unmet need of an unbiased identification and validation of reference genes or miRNAs for exosome studies, here, we performed a sequencing-driven analysis with high-throughput mRNA- and miRNA-Seq datasets from serum exosomes of patients with frequent cancer types and of healthy control individuals and subsequently validate these candidates by qRT-PCR in serum exosomes of an independent cohort of ovarian cancer patients and of healthy control individuals.

## Results

### Identification of candidate reference genes by an unbiased integrative analysis of pooled cancer mRNA-Seq datasets

**Fig. 1:**
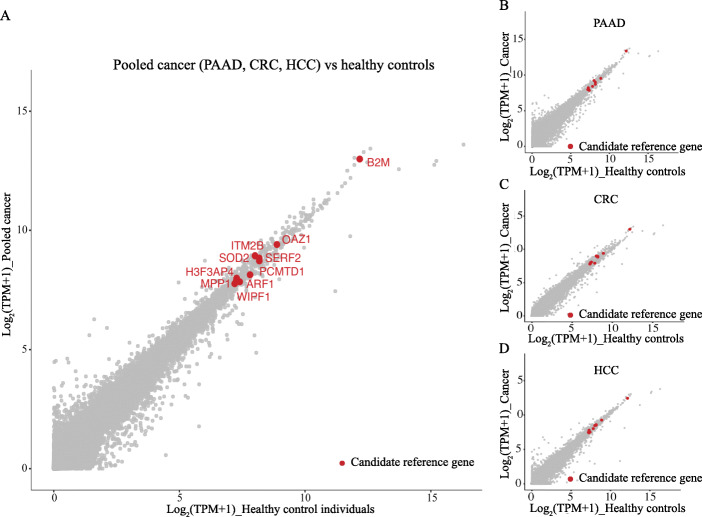
Scatterplots of predicted candidate reference genes for serum exosomes using RNA-Seq data. Expression levels of candidate reference genes in serum exosomes are depicted for pooled cancer samples (PAAD, CRC, HCC) (**a**), for pancreatic adenocarcinoma (PAAD) (**b**), colorectal cancer (CRC) (**c**) and hepatocellular carcinoma (HCC) (**d**) samples and compared to serum exosomes of healthy control individuals. Expression values are shown as the logarithm of transcripts per million (TPM) (log_2_(TPM + 1)). Red dots represent candidate reference genes and grey dots genome-wide genes

To identify reference genes with stable expression in serum exosomes, we interrogated RNA-Seq data from 47 serum exosome samples of patients with PAAD, CRC and HCC as well as of 32 healthy control individuals (HC) and applied Deseq2 to evaluate expression levels across samples. Only genes with high expression in both, serum exosomes of cancer patients and of healthy individuals (measured as transcripts per million (TPM)) compared to the average gene expression level (pooled-transcriptome) were considered as potential reference candidates. Our analysis firstly identified 112, 117, and 85 stably expressed genes respectively in serum exosomes of PAAD, CRC and HCC (*p* value > 0.1), by comparing their patients with healthy control individuals using Deseq2 analysis. Then 48 genes were found to be universally stably expressed in serum exosomes of all cancers. By sorting these genes by their expression level, we identified ten highly expressed candidate reference genes (ADP-ribosylation factor 1 (*ARF1*), beta-2-microglobulin (*B2M)*, H3 histone pseudogene 6 (*H3F3AP4*), integral membrane protein 2B (*ITM2B*), membrane palmitoylated protein 1 (*MPP1*), ornithine decarboxylase antizyme 1 (*OAZ1*), protein-L-isoaspartate (D-aspartate) O-methyltransferase domain containing 1 (*PCMTD1*), superoxide dismutase 2 (*SOD2*), small EDRK-rich factor 2 (*SERF2*), and WAS/WASL Interacting Protein Family Member 1 (*WIPF1*) (Fig. 1a, indicated by red dots and Table 1). The diagonal scatterplot distribution of candidate reference genes indicates consistent expression abundance between exosomes of cancer patients and of healthy control individuals (Fig. 1a), with a correlation coefficient of R = 0.995. Furthermore, expression patterns of candidate reference genes identified by the pooled cancer analysis (including PAAD, CRC and HCC) were recapitulated in each cancer subtype as well (Fig. 1b-d).

**Table 1:** Candidate reference genes (*n* = 10) ranked in order of their expression level and expression stability

Gene Symbol	Expression level		Stability		
	Log_**2**_(TPM + 1)	Rank	CV	M value	Rank
OAZ1	9.289	2	0.405	0.561	1
SERF2	8.608	5	0.492	0.588	2
MPP1	7.748	9	0.460	0.597	3
H3F3AP4	7.750	8	0.654	0.563	4
WIPF1	7.645	10	0.538	0.626	5
PCMTD1	8.067	6	0.511	0.704	6
ARF1	7.850	7	0.660	0.564	7
SOD2	8.696	4	0.655	0.725	8
B2M	12.826	1	0.688	0.827	9
ITM2B	8.754	3	0.723	0.934	10

### Evaluation of expression levels and stability of candidate reference genes

**Fig. 2:**
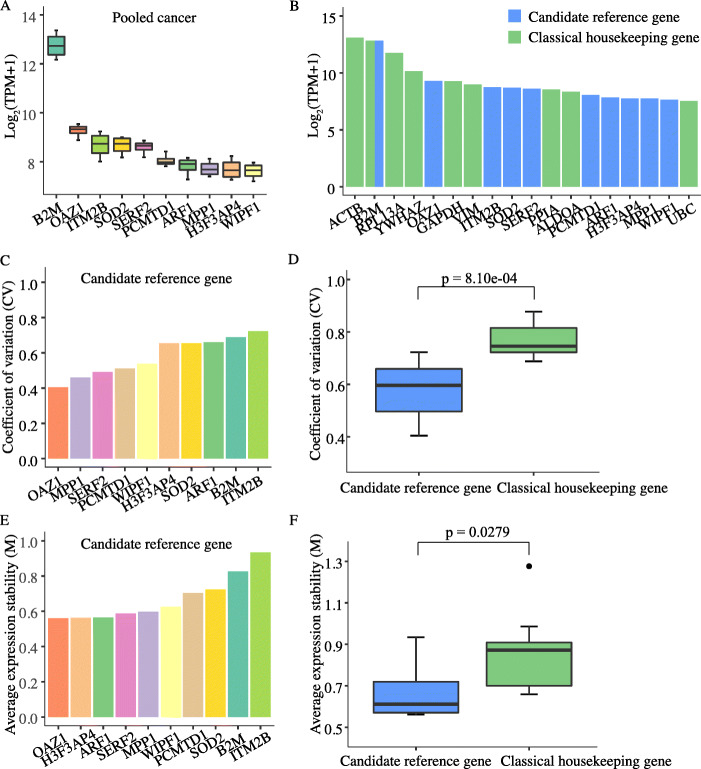
Gene expression levels and stability of candidate reference genes for exosomes predicted with RNA-Seq data. Expression levels of ten candidate genes sorted by their respective expression levels (**a**). Expression levels of ten candidate reference genes (blue bars) compared with those of nine commonly used housekeeping genes (green bars) (**b**). Expression stability of candidate reference genes as measured by the coefficient of variation (“CV”) (**c**). Comparison of “CV” values between candidate reference genes and classical housekeeping genes (*p = 8.10e-04*, Wilcoxon rank-sum test) (**d**). Expression stability of candidate reference genes as measured by the average expression stability value (“M”) (**e**). Comparison of “M” values between candidate reference genes and classical housekeeping genes (*p = 0.0279*, Wilcoxon rank-sum test) (**f**)

To further validate our predicted candidate reference genes for exosomes, we compared their respective expression levels and stabilities with those of nine classical housekeeping genes: beta-actin (*ACTB*), beta-2-microglobulin (*B2M*), ribosomal protein L13A (*RPL13A*), tyrosine 3-monooxygenase/tryptophane 5-monooxygenase activation protein zeta (*YWHAZ*), glyceraldehyde-3-phosphate dehydrogenase (*GAPDH*), vimentin (*VIM*), peptidylprolyl isomerase A (*PPIA*), aldolase A (*ALDOA*), and ubiquitin C (*UBC*). Overall, abundance of exosomal candidate reference genes (Fig. 2a) was similar to those of classical housekeeping genes (Fig. 2b). *B2M* had by far the highest overall expression abundance of all candidate reference genes (Fig. 2a) which was only surpassed by the classical housekeeping gene *ACTB* (Fig. 2b). We then assessed the expression stability across samples and tumor types by two measures: 1) the coefficient of variation “CV” as the standard deviation divided by the mean of the expression levels (transcripts per million - TPM), and 2) the average expression stability “M” determined by the geNorm algorithm. “CV” values for the exosomal candidate reference genes (0.405 to 0.723) (Fig. 2c) were significantly lower than those for classical housekeeping genes (*p = 8.10e-04*, Wilcoxon rank-sum test) (Fig. 2d) with “M” values below 1.0, thus indicating higher expression stability across samples and tumor types (Fig. 2e). The “M” values were also significantly lower in candidate reference genes compared to those for classical housekeeping genes (*p = 0.0279*, Wilcoxon rank-sum test) (Fig. 2f). The candidate reference genes were then sorted according to their expression stability from highest to lowest, and both, the “CV” and “M” criteria achieved similar ranks for most candidates. *OAZ1* was identified as the gene with the highest expression stability across samples and tumor types (Table 1). We also identified and validated ten candidate reference genes respectively for each cancer subtype including PAAD (*FTL*, *OAZ1*, *FYB1*, *SERF2*, *SOD2*, *PCMTD1*, *ARPC2*, *NCOA4*, *HCLS1* and *TYROBP*), CRC (*B2M*, *RPL41*, *SNCA*, *RPS9*, *BTF3*, *ADIPOR1*, *HEMGN*, *SOD2*, *PCMTD1* and *NCOA4*), and HCC (*FTL*, *OAZ1*, *CD74*, *DDX5*, *PCMTD1*, *HCLS1*, *LSP1*, *RPL9*, *WIPF1* and *H3F3AP4*) as well (Suppl. Fig. 1).

### Validation of candidate reference genes in exosomes of an independent cohort of ovarian cancer patients

**Fig. 3:**
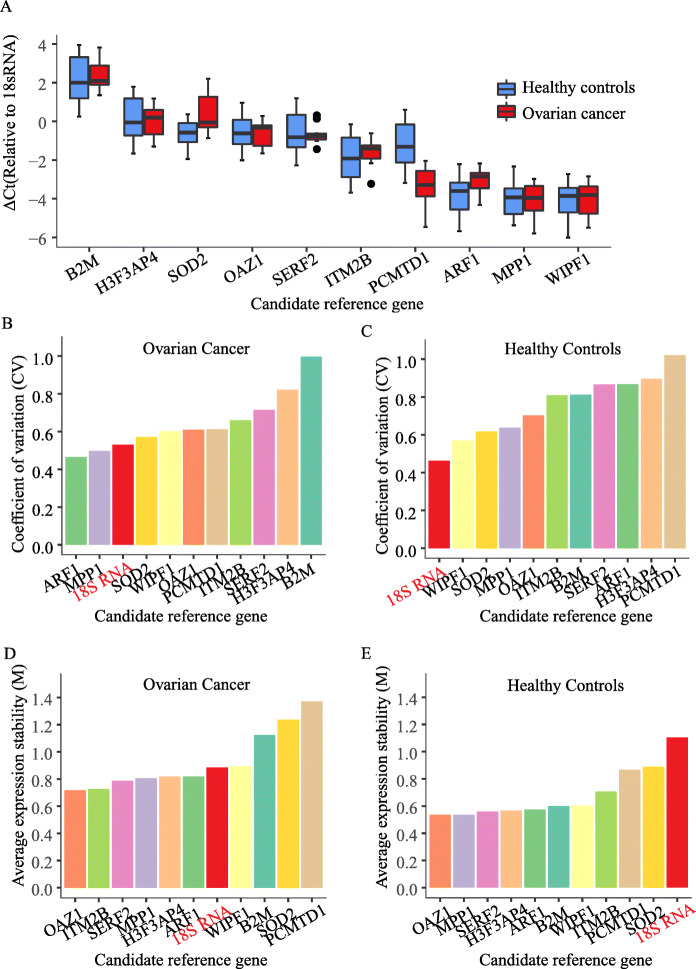
Experimental validation of candidate reference genes in exosomes of patients with ovarian cancer and healthy control individuals. Expression levels (Ct values) of candidate reference genes in exosomes of ovarian cancer patients (red bars) and healthy control individuals (blue bars) relative to *18S rRNA* (**a**). Expression stability of the candidate reference genes in serum exosomes of ovarian cancer patients (**b**) and healthy control individuals (**c**) as measured by the “CV” indicator. Expression stability of the candidate reference genes in serum exosomes of ovarian cancer patients (**d**) and healthy control individuals (**e**) as measured by the “M” indicator

Based on the promising results from the pooled analysis of serum exosomes of patients with different tumour types, we expected our predicted candidate reference genes to be applicable to serum exosomes from patients with various other cancer types as well. Therefore, we next sought to validate the candidate reference genes in a “real-life setting” in samples of serum exosomes of ten ovarian cancer patients and of ten healthy control individuals. The qRT-PCR results showed that as expected from the RNA-Seq data, *B2M* had the highest expression abundance among all candidates (Fig. 3a). Moreover, absolute abundance of *SOD2*, *H3F3AP4*, *OAZ1*, and *SERF2* were comparable to the expression level of *18S rRNA*, whereas the abundance of the remaining five genes (*ITM2B*, *ARF1*, *PCMTD1*, *WIPF1*, *MPP1*) was lower (Fig. 3a). Interestingly, the abundance of the reference candidate genes in serum exosomes of healthy control individuals and of ovarian cancer patients were highly consistent (Fig. 3a). Most candidate genes also exhibited high expression stability in ovarian cancer and healthy control individuals with “M” and “CV” values lower than 1.0 (Fig. 3b-e), even though some variation occurred regarding the gene order between both stability indicators. Whereas *MPP1*, *WIPF1*, *SOD2* and *OAZ1* exhibited lower “CV” values in exosomes of healthy individuals (Fig. 3c), in both exosome groups, *OAZ1* had the lowest “M” value (Fig. 3d-e). The “M” values for *OAZ1*, *ITM2B*, *SERF2*, *MPP1*, *H3F3AP4*, and *ARF1* were advantageous over *18S rRNA*, whereas *WIPF1*, *B2M*, *SOD2* and *PCMTD1* in part had clearly higher “M” values indicating reduced expression stability (Fig. 3d). The expression stability of *18S rRNA* was lower (indicated by a higher “M” value”) compared to many of the identified candidate reference genes especially in exosomes of healthy control individuals (Fig. 3d-e).

To quantify gene expression levels more accurately, multiple reference genes can be used [46]. Therefore, we also determined the expression stability of respective combinations of candidate reference genes by determining the average gene-specific variation with the geNorm algorithm for RNA-Seq datasets in exosomes of the pooled cancer populations and for qRT-PCR data of exosomes from ovarian cancer patients. Overall, three combinations according to their expression stability ranks (Table 1) were evaluated: 1) genes 1–3 (*OAZ1*, *SERF2*, *MPP1*); 2) genes 4–6 (*H3F3AP4*, *WIPF1*, *PCMTD1*); and 3) genes 8–10 (*SOD2*, *B2M*, *ITM2B*). The first group with a combination of *OAZ1*, *SERF2* and *MPP1* had the lowest average gene-specific variations in exosomes of the pooled patient group including PAAD, HCC and CRC (RNA-Seq, Suppl. Fig. 2A) as well as in ovarian cancer patients (qRT-PCR, Suppl. Fig. 2B) indicating the highest expression stability.

### Identification and validation of candidate reference miRNAs in cancer exosomes

**Fig. 4:**
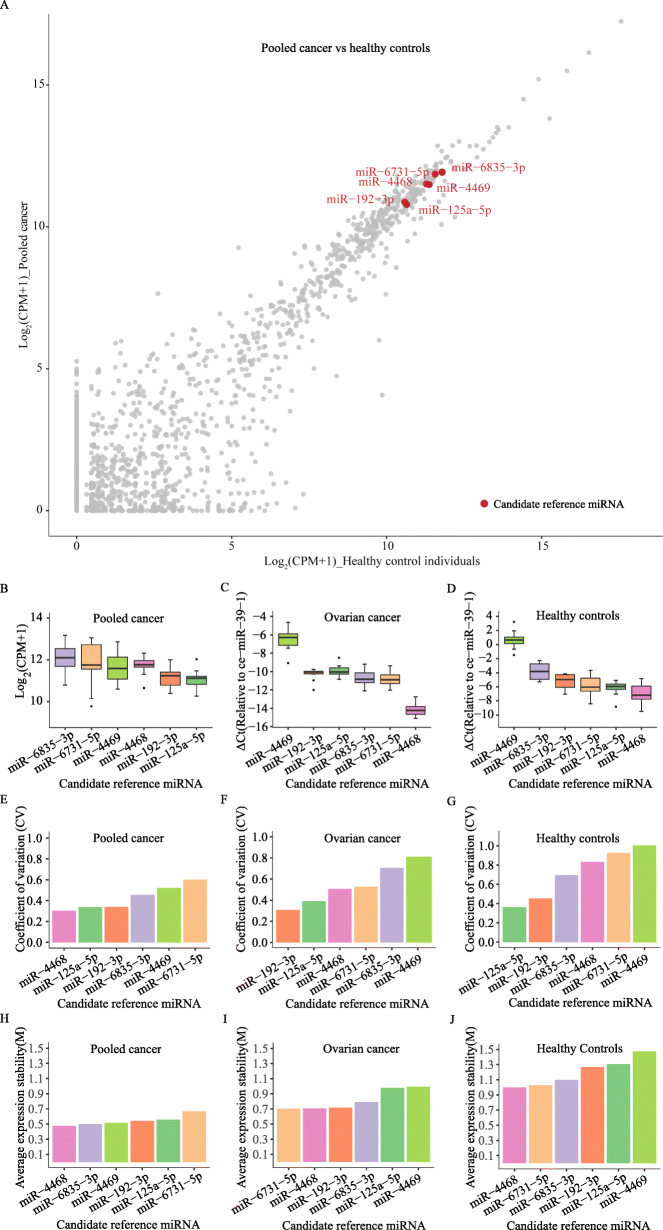
Identification and validation of candidate reference miRNAs predicted in exosomes of ovarian cancer patients. Scatterplot of candidate reference miRNA expression levels in pooled cancer samples (HCC, HNSCC, LCA, NBL, OVA, and THCA) and healthy control individuals. Expression values are shown as the logarithm of counts per million (CPM) (log_2_(CPM + 1)). The red dots represent candidate reference miRNAs, grey dots genome-wide miRNAs (**a**). Expression levels of six candidate reference miRNAs in exosomes of pooled cancer (**b**), ovarian cancer patients (relative to ce-miR-39-1, n = 10) (**c**) and healthy control individuals (relative to ce-miR-39-1, n = 10) (**d**). Expression stability of candidate reference miRNAs in exosomes of pooled cancer (**e**), ovarian cancer patients (**f**) and healthy control individuals (**g**) as measured by the “CV”. Expression stability of six candidate reference miRNAs in exosomes of pooled cancer (**h**), ovarian cancer patients (**i**) and healthy control individuals (**j**) as measured by the “M” indicator

In addition to mRNA, exosomes also contain miRNA. To identify reliable miRNAs for normalization in exosomes, we analyzed miRNA-Seq data of 72 serum exosome samples of patients with HCC, HNSCC, LCA, NBL, OVA, and THCA and 31 serum exosome samples of healthy control individuals. We identified six candidate reference miRNAs with high and stable expression: hsa-*miR-125-5p, hsa-miR-192-3p*, *hsa-miR-4468*, *hsa-miR-4469, hsa-miR-6731-5p*, and *hsa-miR-6835-3p* (Fig. 4a). Expression levels and stability of the candidate reference miRNAs were evaluated in the exosomes of pooled cancer and further validated in the exosomes of ovarian cancer and healthy control individuals (Fig. 4b-j). Across the pooled exosomes of six cancer types, but also for each individual cancer type, these candidate miRNAs show high expression and similar abundance compared to exosomes of healthy control individuals (depicted as counts per million (CPM)) (Fig. 4b, Suppl. Fig. 3). Among all candidate miRNAs, *hsa-miR-6835-3p* had the highest expression level across samples and tumor types (Table 2). And *hsa-miR-4468* had the highest and *hsa-miR-6731-5p* the lowest expression stability across samples and cancer types as indicated by low and high “CV” and “M” values, respectively (Fig. 4e, h). Overall, “M” values for all candidate miRNAs were low (< 1.5), indicating their general expression stability and potential utility as candidate reference miRNAs for exosomes. By integrating both stability indicators “CV” and “M”, candidate reference miRNAs were ranked and hsa-miR-4468 showed the highest overall expression stability across samples and tumor types (Table 2). Finally, *hsa-miR-6835-3p with* high expression level and stability was identified as the best reference miRNA.

**Table 2:** Candidate reference miRNAs (*n* = 6) ranked in order of their expression level and expression stability

miRNA	Expression level		Stability		
	Log_**2**_(CPM + 1)	Rank	CV	M value	Rank
hsa-miR-4468	11.743	4	0.476	0.304	1
hsa-miR-6835-3p	12.208	1	0.498	0.455	2
hsa-miR-192-3p	11.223	5	0.543	0.340	3
hsa-miR-125a-5p	11.102	6	0.557	0.336	4
hsa-miR-4469	11.756	3	0.515	0.521	5
hsa-miR-6731-5p	12.087	2	0.667	0.601	6

To further validate the predicted reference miRNA candidates, we measured their expression levels by qRT-PCR in serum exosomes of patients with ovarian cancer (*n* = 10) and of healthy control individuals (n = 10). miRNA abundance was calculated as cycle threshold numbers (Ct) relative to *ce-miR-39-1*. *ce-miR-39-1* is a frequently used miRNA for normalization (Fig. 4c-d). These results showed the highest expression for *hsa-miR-4469* in exosomes of ovarian cancer patients even though all miRNAs were less abundant than *ce-miR-39-1* (Fig. 4c-d). In exosomes of ovarian cancer patients, *hsa-miR-4469* and *hsa-miR-4468* were the miRNAs with the highest and lowest expression levels*,* reproducing the results for exosomes of healthy control individuals (Fig. 4c-d). Compared to the miRNA-Seq analysis (Fig. 4e, h), *hsa-miR-6731-5p*, *hsa-miR-4468*, *hsa-miR-192-3p* and *hsa-miR-6835-3p* exhibited lower “CV” and “M” values indicating even higher expression stability in a “real-life” setting (Fig. 4f, g, i, j). Overall, all candidate reference miRNAs in exosome of ovarian cancer and healthy control individuals exhibited “M” values smaller than 1.5 indicating high expression stability (Fig. 4i-j).

Furthermore, the expression stability of combinations of multiple reference miRNAs was determined by the average gene-specific variation. We generated three combinations of two candidate reference miRNAs each according to their expression stability ranks (Table 2): 1) miRNAs 1–2 (*hsa-miR-4468* and *hsa-miR-6835-3p*), 2) miRNAs 3–4 (*hsa-miR-192-3p* and *hsa-miR-125a-5p)*, and 3) miRNAs 5–6 (*hsa-miR-4469* and *hsa-miR-6731-5p*). The combination of *hsa-miR-6835-3p* and *hsa-miR-4468* had the highest expression stability in exosomes of pooled groups of patients affected by PAAD, HCC and CRC (miRNA-Seq data, Suppl. Fig. 4A) or by ovarian cancer (qRT-PCR data, Suppl. Fig. 4B).

## Discussion

Exosomes are nano-sized (< 200 nm in diameter) biovesicles which are released into the surrounding body fluids upon fusion of endocytic compartments with the plasma membrane [47] . Exosomes transfer various types of cargo from donor to acceptor cells among them nucleic acids, mRNAs and miRNAs were the first nucleic acids to be identified in exosomes [3]. Interestingly, some mRNAs and miRNAs are even specifically enriched in cancer exosomes implying a critical role for cancer biology and progression. Therefore, exosomes can be harnessed as diagnostic biomarkers or as targets for therapeutic interventions [3, 5, 48–50]. To characterize the composition of exosomes, the accurate quantification of mRNA and miRNA expression within the exosome fraction is critical. qRT-PCR combines high sensitivity and specificity with high reproducibility and low template input requirements and is therefore an ideal technology for exosome studies [22, 23]. qRT-PCR analyses, however, require the selection of appropriate reference genes to avoid variation in gene expression results under different experimental conditions (e.g. tumor cell vs. exosome) [22, 30, 31, 51] and currently, the ideal reference genes for the analysis of exosomes across cancers or for comparison of expression with cancer cells or tissues remain largely unknown [52, 53]. Often, classical housekeeping genes used for gene expression analyses in tissues or cell lines are used for exosome studies as well, but the expression stability of these genes is not unconditionally guaranteed for exosome samples thereby limiting the analytical accuracy. In this context, previous studies have confirmed that there is no universal reference gene for normalization under different conditions [35, 36, 54, 55].

Therefore, here, we sought to perform an unbiased and sequencing-driven analysis of publicly available high-throughput RNA- and miRNA-Seq datasets to identify and experimentally validate reference genes and combinations of these genes to generate accurate RNA- and miRNA-expression data from serum exosomes of cancer patients. From the pooled RNA- and miRNA-Seq datasets we identify multiple potential candidate reference genes in cancer exosomes (*ARF1*, *B2M*, *H3F3AP4*, *ITM2B*, *MPP1*, *OAZ1*, *PCMTD1*, *SOD2*, *SERF2*, *WIPF1* for mRNA analyses and *hsa-miR-125-5p, hsa-miR-192-3p*, *hsa-miR-4468*, *hsa-miR-4469, hsa-miR-6731-5p*, and *hsa-miR-6835-3p* for miRNA analyses) (Tables 1 and 2 and Suppl. Table 1) and subsequently validate their expression stability in exosomes isolated from sera of patients with ovarian cancer and of healthy control individuals. All ten identified candidate reference genes provide better accuracy in terms of stability and variation of expression compared to classical housekeeping genes (Table 1, Fig. 2d and f). Interestingly, if we applied our algorithm to exosome data of each individual cancer type, the predicted candidate reference genes were different from those in the pooled cancer analysis and also varied among cancer subtypes (Suppl Fig. 1).

By employing two different indicators, we define mRNA and miRNA expression stability from two different perspectives: 1) the coefficient of variation “CV” and 2) the “M” value which are both based on the expression abundance of a gene (measured as transcripts per million – TPM) or miRNA (measured as counts per million – CPM). Whereas “CV” measures the variation of a reference gene across all samples, “M” represents the average pairwise variation of a reference gene versus all other reference genes across all samples. Low “CV” and “M” values suggest stable gene expression, and in general, genes with “M” < 1.5 can be accepted as reference genes [54]. By requiring a reference gene to have ideally both, low “CV” and “M” values, we identify *OAZ1* and *hsa-miR-6835-3p* as the most accurate single reference genes based on RNA-Seq datasets (Tables 1 and 2). We confirm the utility of all candidate reference genes and miRNAs but especially of *OAZ1* and *hsa-miR-6835-3p* by measuring their expression abundance (Fig. 3a and Fig. 4c, d) and stability (Fig. 3b, c, d, e and Fig. 4f, g, i, j) in serum exosomes of an independent cohort of ovarian cancer patients and of healthy control individuals (*n* = 10 each group, Fig. 3). NormFinder [56] is another popular method without requiring priori knowledge of control gene as calibrator to enhance the accuracy, by calculating intra and intergroup variations to evaluate the stability of expression. Here we used NormFinder to evaluate the candidates predicted by geNorm. The results (Suppl. Fig. 5) showed that the most reliable genes *OAZ1* and *hsa-miR-6835-3p* still presented a robust stability measured by NormFinder, with *OAZ1* ranking the 1st in healthy controls and the 2nd in ovarian cancer patients (Suppl. Fig. 5A, B), and with *hsa-miR-6835-3p* ranking the 1st in healthy controls and the 3rd in ovarian cancer (Suppl. Fig. 5C, D). To increase the diagnostic accuracy, some studies suggest the use of a combination of multiple reference genes or miRNAs [52, 54, 57]. We therefore tested various combinations of reference gene candidates, and identified the combinations of *OAZ1*, *SERF2* and *MPP1* for mRNA (Suppl. Fig. 2) and *hsa-miR-6835-3p* and *hsa-miR-4468* for miRNA (Suppl. Fig. 4) providing the highest expression stability across samples and tumor types.

Unlike many previous studies which used an approach to narrow down the number of reference genes from a panel of previously reported candidate genes - usually a list of classical housekeeping genes [37, 45, 58, 59] - our study has the clear advantage of an unbiased and sequencing data-driven approach thus preventing bias from artificial selection. Due to the scarcity of publicly available RNA- and miRNA-Seq exosome datasets with high sequencing quality, the exosome datasets used herein include the RNA-Seq exosome samples with PAAD, CRC and HCC as well as miRNA-Seq exosome samples with HCC, HNSCC, LCA, NBL, OVA, and THCA. Accounting for the limitation that the samples in our current analysis may not fully capture the dynamic expression of genes in exosomes of pan-cancers, our analysis will be continuously updated with the emergence of additional sequencing datasets to refine the robustness of identified candidate reference genes. In this regard, it will be interesting to determine if the reference genes and miRNAs identified here will also proof utility in a true pooled cancer analysis including most or even all of the different cancer types affecting patients.

In our analysis, pooled cancer RNA- or miRNA-Seq datasets are from the extracellular vesicles (including exosomes) isolated from the different labs. However, different sized vesicles are derived from different intracellular processes, that affects the numbers and biomolecule contents. Other technical factors (such as exosome isolation and quantification procedures) and biological factors (such as cancer type) can also impact the numbers and composition of exosomes [60–62]. Exosomes isolated from the different sources or by the different isolation methodologies introduce variations in the concentration, purity and size [28, 29]. Moreover, due to the limited knowledge of exosome specific molecular machineries of biogenesis and release, there are the challenges in confirming the biogenesis mechanisms of exosomes. Therefore, future efforts should definitely include standardization (number, volume, etc) and verification of the exosome or extracellular vesicle characteristics, prior to sequencing.

We finally confirm previous study results indicating, that housekeeping genes in cancer cell lines and tumor tissue cannot be transferred to the analysis of exosomes and vice versa without further validation [22, 31, 35, 36]. For this, we predicted the top ten candidate reference genes (*ACTB*, *RPS27*, *RPS11*, *RPL13A*, *RPL41*, *RPS14*, *RPL41P1*, *RPS29*, *RPL10* and *NACA*), in cancer tissues with simultaneous high expression abundance (log_2_(TPM + 1)) and stable expression (“CV” and “M”) (Suppl. Fig. 6A), and compared their ranks with those of the top ten predicted reference genes in exosomes and vice versa (Suppl. Fig. 6B). The top ten genes with the high expression stability in tissues as indicated by low “CV” and “M” values were ranked the 120th to 600th in exosomes (Suppl. Fig. 6B, C, D). The results clearly show that there is no overlap between candidate reference genes in exosomes and cancer cell lines/tissue if both, exosome and cancer cell line datasets are not considered together in the initial step of candidate reference prediction (Suppl. Fig. 6B). Although derived from tissue, exosomes deliver specific cargo of protein, miRNA, and small molecules, which is heterogenous between exosome and tissue. The use of tissue-specific reference genes causes quantitative inaccuracy due to the instability of them in exosome studies. Therefore, it is great practical necessity for use exosome-specific reference genes to enhance the quantity accuracy.

## Conclusions

Our study provides reference genes and miRNAs with high abundance and expression stability across samples and cancer types for more accurate future qRT-PCR analyses of cancer exosomes. *OAZ1* and *hsa-miR-6835-3p* were identified as the most reliable individual reference genes for mRNA and miRNA quantification, respectively. For superior accuracy, we recommend the use of a combination of reference genes - *OAZ1*/*SERF2*/*MPP1* for mRNA and *hsa-miR-6835-3p*/*hsa-miR-4468-3p* for miRNA analyses. The use of the ideal reference genes is favorable to the accurate quantification of mRNA and miRNA expression fraction within the exosome.

## Methods

### Data collection

We manually curated RNA- and miRNA-Seq exosome data from the NCBI Gene Expression Omnibus (GEO; https://www.ncbi.nlm.nih.gov/geo/) [63]. The RNA-Seq datasets included 79 serum exosome samples from patients with pancreatic adenocarcinoma (PAAD), colorectal cancer (CRC), hepatocellular carcinoma (HCC) and healthy control individuals (HC) (Suppl. Table 2). The miRNA-Seq datasets included 72 serum exosome samples from patients with seven different cancer types (HCC, lung cancer (LCA), ovarian cancer (OVA), head and neck squamous cell carcinoma (HNSCC), neuroblastoma (NBL) and thyroid cancer (THCA)) as well as healthy control individuals (*n* = 31) (Suppl. Table 2). For comparison of expression patterns between exosomes and cancer tissues or cell lines, we furthermore analyzed RNA-Seq data from 28 datasets including three cancer types (PAAD, CRC, and HCC) with overall 1038 tissue and cancer cell line samples (Suppl. Tab. 2). To validate the reference genes identified by mRNA- and miRNA-Seq analyses, the serum samples were collected from 10 patients diagnosed with ovarian cancer stage I and II according to TNM staging without any treatment and 10 healthy volunteers were recruited in the study.

### Processing of raw RNA- and miRNA-Seq data

Raw reads of the RNA-Seq data were trimmed by removing adaptors and low-quality bases using Trimmomatic (version 0.36) [64]. The clean reads were then mapped onto a human reference genome (GRCh38) using HASAT2 software (version 2.1.0) [65]. StringTie (version 1.3.0) [66] was applied to quantify the number of reads that were aligned to the regions of protein-coding RNAs (mRNAs) and annotations of mRNAs in the human genome were retrieved from GENCODE (v29) [67]. For the identification of reference miRNAs, sequencing data were additionally discarded of low-quality reads, adaptor dimers and sequences with lengths < 18 and > 35 nucleotides. The filtered reads were mapped to the human genome by bowtie (version 1.2.1.1) [68] and quantified by featureCounts (version 1.5.3) [69], miRNA annotations were retrieved from miRBase (v22.1) [70]. Expression levels were depicted as transcripts per million for mRNA (TPM) [71] and counts per million (CPM) for miRNA.

### Strategy to identify candidate reference genes and miRNAs in cancer exosomes

For individual cancer subtypes, we selected genes or miRNAs as reference candidates if the respective expression level was greater than the average genome-wide expression. Therefore, genes or miRNAs were analyzed using DESeq2 (version 1.22.1) in serum exosomes of cancer patients and of healthy control individuals [72]. DESeq2 provides algorithms for determining differential expression within digital expression datasets using a negative binomial distribution model. In the present study, genes with a *p*-value > 0.1 were considered to be not differentially expressed.

To determine the expression stability of candidate reference genes and of miRNAs across samples and cancer types, we used two measures: 1) “CV” - the variation of a candidate reference gene or miRNA across all samples - and 2)” M” - the average expression stability value which represents the average pairwise variation between a reference gene and other reference genes across samples calculated by geNorm [54]. Lower values of “CV” or “M” indicate higher expression stability.

We assumed that there were *m* samples and *n* reference genes. For a given reference gene *j* in the *ith* sample, the gene expression level *a*_*ij*_ is the normalization expression value (TPM for mRNA, CPM for miRNA) of RNA−/miRNA-Seq data or of the transformed value of cycle threshold numbers (Ct) in qRT-PCR data (Eq. 1). Based on the expression levels of the reference gene *j* across *m* samples (Eq. 2), we defined the coefficient of variation “CV” as the ratio of the standard deviation to the mean (Eq. 3).

$$ \left(\forall i\in \left[1,m\right],\forall j\in \left[1,n\right]\right): $$

1 $$ {a}_{ij}={2}^{-{C}_{t_{ij}}} $$

2 $$ {A}_j=\left({a}_{1j},{a}_{2j},\dots {a}_{mj}\right)={\left({a}_{ij}\right)}_{i=1\to m} $$

3 $$ {CV}_j=\frac{st. dev\left({A}_j\right)}{mean\left({A}_j\right)} $$

The second expression stability measure - the average expression stability value “M” - was developed by Vandesompele J et al. in the tool geNorm [54]. For any two reference genes *j* and *k*, the logarithm of the expression ratio $$ \raisebox{1ex}{${a}_{ij}$}\!\left/ \!\raisebox{-1ex}{${a}_{ik}$}\right. $$ for the sample *i* (*i* = 1 → *m*) forms an array *A*_*jk*_ (Eq. 4). Based on the pairwise variation *V*_*jk*_ defined by the standard deviation of *A*_*jk*_ (Eq. 5), the average expression stability value *M*_*j*_ for the gene *j* is the arithmetic mean of all pairwise variation *V*_*jk*_ (Eq. 6). Usually, a gene with a *M* less than 1.5 is acceptable as a reference gene.

$$ \left(\forall j,k\in \left[1,n\right]\  and\ j\ne k\right): $$

4 $$ {A}_{jk}=\left\{{\log}_2\left(\frac{a_{1j}}{a_{1k}}\right),{\log}_2\left(\frac{a_{2j}}{a_{2k}}\right),\dots {\log}_2\left(\frac{a_{mj}}{a_{mk}}\right)\right\}={\left\{{\log}_2\left(\frac{a_{ij}}{a_{ik}}\right)\right\}}_{i=1\to m} $$

5 $$ {V}_{jk}= st. dev\left({A}_{jk}\right) $$

6 $$ {M}_j=\frac{\sum \limits_{k=1}^n{V}_{jk}}{n-1} $$

Furthermore, multiple genes were combined as candidate references and their stability was measured as the average gene-specific variation *AV* according to geNorm. For any three reference genes *j*, *k* and *l*, the average gene-specific variation *AV*_*j*, *k*, *l*_ was calculated as the geometric mean of the three-gene stability value “*M*" (Eq. 7).

$$ \left(\forall j,k,l\in \left[1,n\right]\  and\ j\ne k\ne l\right): $$

7 $$ {AV}_{j,k,l}=\sqrt[3]{M_j\bullet {M}_k\bullet {M}_l} $$

### Isolation of the exosome fraction and sample storage

To validate the reference genes identified by mRNA- and miRNA-Seq analyses, serum exosomes were obtained from ten ovarian cancer patients and ten healthy control individuals. Therefore, blood was drawn and kept at room temperature (15–25 °C) for 10 min to 1 h before further processing. The tubes were centrifuged for 10 min at 1900 x g (3,000 rpm) and 4 °C using a swinging bucket rotor. The upper serum phase was transferred into a new canonical tube without disturbing the cell pellet. Subsequently, sera were centrifuged for 15 min at 3000 x g and 4 °C in conical tubes, passed through a 0.8-μm filter and the supernatants were carefully removed without disturbing the pellet and transferred into new tubes. Samples were stored at 2–8 °C for immediate processing or kept frozen in aliquots at − 65 °C to − 90 °C for long-term storage [29].

### Quantitative reverse transcription-PCR (qRT-PCR)

Total RNA (containing the mRNA and miRNA fractions) was isolated from 1 ml serum using the exoRNeasy Serum/Plasma Maxi Kit (Qiagen, Wetzlar, Germany). cDNA was generated with the PrimeScriptRT Master Mix (Perfect Real Time; Takara Bio, Kusatsu, Japan) according to the manufacturer’s protocol. Quantitative RT-PCR was performed with ChamQTM Universal SYBR qPCR Master Mix (Vazyme, Nanjing, China) in a final reaction volume of 10 μl using an Applied Biosystems QuantStudio 5 Real-Time PCR Instrument (Thermo Fisher Scientific, Rockford, USA). *18S rRNA* and ce-miR-39-1 served as internal controls for mRNA and miRNA, respectively. Expression levels are depicted as cycle threshold (Ct) value of the candidate gene relative to the Ct value of the housekeeping gene. Data were analyzed with the QuantStudio TM Design & Analysis software. The primer sequences for B2M were 5′-TGTCTTTCAGCAAGGACTGGT-3′ and 5′-TGCTTACATGTCTCGATCCCAC-3′, for OAZ1 were 5′-GCCAAACGCATTAACTGGCG-3′ and 5′-TGTCCTCGCGGTTCTTGTG-3′, for ITM2B were 5′-TTGCCTCAGTCCTATCTGATTCA-3′ and 5′-TCTGCGTTGCAGTTTGTAAGT-3′, for SOD2 were 5′-GGAAGCCATCAAACGTGACTT-3′ and 5′-CCCGTTCCTTATTGAAACCAAGC-3′, for PCMTD1 were 5′-TGCATTTGTTGTTGGTAATTGCC-3′ and 5′-GTCCAGTTCGCATAATCTGTGT-3′, for ARF1 were 5′-ATGGGGAACATCTTCGCCAAC-3′ and 5′-GTGGTCACGATCTCACCCAG-3′, for MPP1 were 5′-GTCAGCTCCTAGCGAAGCC-3′ and 5′-GCCGAACGACTTCCTCGTAG-3′, for WIPF1 were 5′-AGCCGCTGCGCGATTTAT-3′ and 5′-TCCCAGCCTGCTCTGTCTTA-3′, for SERF2 were 5′-CCGCAAGCAGAGGGACTC-3′ and 5′-AGCACTACAGGAGGAAACGC-3′, for H3F3AP4 were 5′-CAGCTATCGGTGCTTTGCAG-3′ and 5′-AGCACGTTCTCCACGTATGC-3′, for 18 s were 5′-CTTCCACAGGAGGCCTACAC-3′ and 5′-CTTCGGCCCACACCCTTAAT-3′.

### Statistical analysis

The Wilcoxon rank-sum test was used to compare expression stability between classical housekeeping and exosomal candidate reference genes as measured by “CV” and “M”. Candidate reference genes were sorted according to their “CV” and “M” values from low (higher expression stability across samples) to high (lower expression stability across samples) and assigned a rank, and the best candidate gene or miRNA for validation was determined as the one with the lowest sum of these two ranks. All statistical analyses were executed in R.

## Supplementary Information

**Additional file 1: Figure S1.** Gene expression level and stability of candidate reference genes in PAAD, HCC and CRC exosomes predicted using RNA-Seq data **A**, Expression levels of ten candidate genes in PAAD (**A**), HCC (**B**) and CRC (**C**) exosomes sorted by their expression. Expression stability of ten candidate reference genes in exosomes of patients with PAAD (**D**), HCC (**E**) and CRC (**F**) measured by the “CV”. Expression stability of ten candidate reference genes in exosomes of PAAD (**G**), HCC (**H**) and CRC (**I**) patients as measured by the “M” indicator. Expression levels are given as the log_2_(TPM + 1) (TPM – transcripts per million). The TPMs of each candidate gene were used to determine “CV” and “M” indicators.

**Additional file 2: Figure S2.** Evaluation of expression stability of combinations of three reference genes in pooled exosomes of cancer patients with PAAD, CRC and HCC (A, RNA-Seq data) and with ovarian cancer (B, qRT-PCR data) The expression stability of respective combinations was measured as the average gene-specific variation calculated with the geNorm algorithm based on transcripts per million (TPM) (**A**) or cycle threshold (Ct) values (**B**). Three combinations according to their expression stability ranking from Table 1 were evaluated: 1) genes 1–3 (*OAZ1*, *SERF2*, *MPP1*); 2) genes 4–6 (*H3F3AP4*, *WIPF1*, *PCMTD1*); and 3) genes 8–10 (*SOD2*, *B2M*, *ITM2B*)

**Additional file 3: Figure S3.** Scatterplots of expression levels of six candidate reference miRNAs (red dots) in serum exosomes of patients with HCC (**A**), HNSCC (**B**), LCA (**C**), NBL (**D**), OVA (**E**) and THCA (**F**) compared to exosomes of healthy control individuals The expression values are depicted as: log_2_(CPM + 1) (CPM – counts per million). Grey dots indicate genome-wide miRNAs

**Additional file 4: Figure S4.** Evaluation of expression stability of combinations of two reference miRNA candidates in pooled exosomes of cancer patients with different tumor types (**A**, miRNA-Seq data) or with ovarian cancer (**B**, qPCR data) The expression stability of miRNA combinations was measured as the average miRNA-specific variation, which was calculated by the geNorm algorithm based on counts per million (CPM) (**A**) or cycle threshold (Ct) values (**B**). Three combinations were considered according to the miRNA expression stability ranks shown in Table 2: miRNAs 1–2 (*miR-4468* and *miR-6835-3p*), miRNAs 3–4 (*miR-192-3p* and *miR-125a-5p)*, and miRNAs 5–6 (*miR-4469* and *miR-6731-5p*)

**Additional file 5: Figure S5.** Validation of candidate reference genes and miRNAs predicted in exosomes of ovarian cancer patients and healthy controls by NormFinder Expression stability of candidate reference genes as measured by the NormFinder stability value in exosomes of ovarian cancer patients (**A**) and healthy control individuals (**B**). Expression stability of six candidate reference miRNAs in exosomes of ovarian cancer patients (**C**) and healthy control individuals (**D**) as measured by the NormFinder stability value

**Additional file 6: Figure S6.** Analysis of candidate reference genes predicted in cancer tissues Expression levels of the ten top candidates in pooled cancer tissue samples calculated using RNA-Seq data. Expression levels are given as log_2_(TPM + 1; TPM = transcript per million) (**A**). The top ten predicted candidate reference genes for exosomes were compared with the respective ranking in cancer tissues and vice versa (**B**). Expression stability of the ten top candidate reference genes in tumor tissues as measured by “CV” (**C**) and “M” indicators (**D**)

**Additional file 7: Table S1.** List of candidate reference genes (*n* = 10) and miRNAs (*n* = 6) identified by RNA-Seq and quantitative real-time PCR analyses.

**Additional file 8: Table S2.** Detailed information for the RNA-Seq datasets in the GEO database.

## Data Availability

The datasets analysed during the current study are available in the Gene Expression Omnibus (GEO), [https://www.ncbi.nlm.nih.gov/geo/]. The datasets information is included in the supplementary materials.

## Abbreviations

EVs: Extracellular vesicles
mRNA: Messenger RNA
miRNA: MicroRNA
RNA: Ribonucleic acid
RNA-Seq: RNA-sequencing
miRNA-Seq: MicroRNA-sequencing
qRT-PCR: Quantitative real-time polymerase chain reaction
TPM: Transcripts per million
CPM: Counts per million
Ct: Cycle threshold numbers
CV: Coefficient of variation
PAAD: Pancreatic adenocarcinoma
CRC: Colorectal cancer
HCC: Hepatocellular carcinoma
HC: Healthy control individuals
LCA: Lung cancer
OVA: Ovarian cancer
HNSCC: Head and neck squamous cell carcinoma
NBL: Neuroblastoma
THCA: Thyroid cancer
*B2M*: Beta-2-microglobulin
*ARF1*: ADP-ribosylation factor 1
*H3F3AP4*: H3 histone pseudogene 6
*ITM2B*: Integral membrane protein 2B
*MPP1*: Membrane palmitoylated protein 1
*OAZ1*: Ornithine decarboxylase antizyme 1
*PCMTD1*: Protein-L-isoaspartate (D-aspartate) O-methyltransferase domain containing 1
*SOD2*: Superoxide dismutase 2
*SERF2*: Small EDRK-rich factor 2
*WIPF1*: WAS/WASL Interacting Protein Family Member 1
*ACTB*: Beta-actin
*RPL13A*: ribosomal protein L13A
*YWHAZ*: Tyrosine 3-monooxygenase/tryptophane 5-monooxygenase activation protein zeta *GAPDH* glyceraldehyde-3-phosphate dehydrogenase
*VIM*: Vimentin
*PPIA*: Peptidylprolyl isomerase A
*ALDOA*: Aldolase A
*UBC*: Ubiquitin C
*FTL*: Ferritin light chain,
*FYB1*: FYN binding protein 1
*ARPC2*: Actin related protein 2/3 complex subunit 2
*NCOA4*: Nuclear receptor coactivator 4
*HCLS1*: Hematopoietic cell-specific Lyn substrate 1
*TYROBP*: Transmembrane immune signaling adaptor TYROBP
*RPL41*: Ribosomal protein L41
*SNCA*: Synuclein alpha
*RPS9*: Ribosomal protein S9
*BTF3*: Basic transcription factor 3
*ADIPOR1*: Adiponectin receptor 1
*HEMGN*: Hemogen
*CD74*: CD74 molecule
*DDX5*: DEAD-box helicase 5
*LSP*: Lymphocyte specific protein 1
*RPL9*: Ribosomal protein L9
