# Supplementary figures and images for: Unbiased RNA-Seq-driven identification and validation of reference genes for quantitative RT-PCR analyses of pooled cancer exosomes
Source: BMC Genomics. 2021 Jan 6;22:27. doi: 10.1186/s12864-020-07318-y (PMC7789813; doi:10.1186/s12864-020-07318-y)

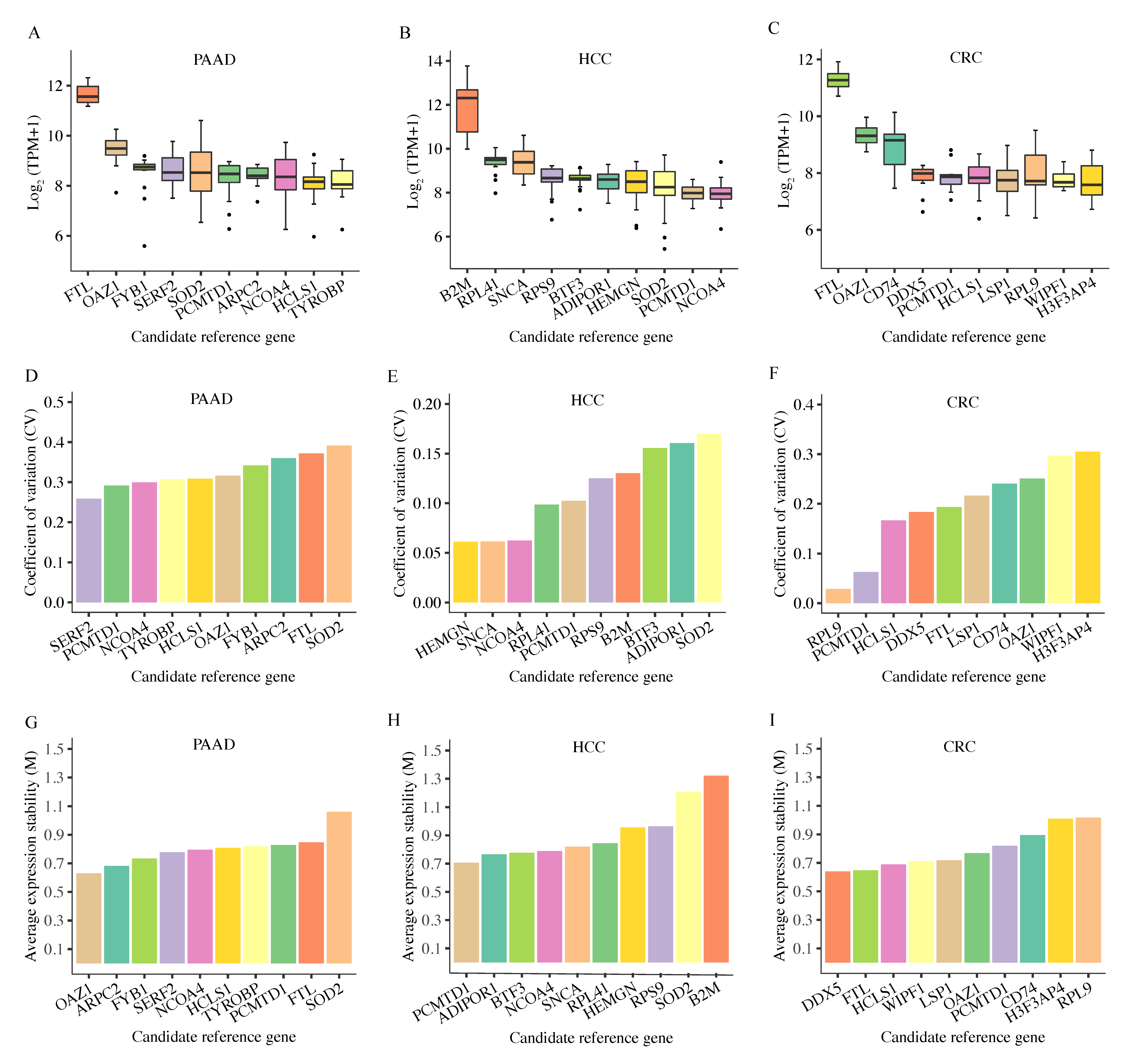

Supplement: Supplementary file 1 — Additional file 1: Figure S1. Gene expression level and stability of candidate reference genes in PAAD, HCC and CRC exosomes predicted using RNA-Seq data A, Expression levels of ten candidate genes in PAAD (A), HCC (B) and CRC (C) exosomes sorted by their expression. Expression stability of ten candidate reference genes in exosomes of patients with PAAD (D), HCC (E) and CRC (F) measured by the “CV”. Expression stability of ten candidate reference genes in exosomes of PAAD (G), HCC (H) and CRC (I) patients as measured by the “M” indicator. Expression levels are given as the log2(TPM + 1) (TPM – transcripts per million). The TPMs of each candidate gene were used to determine “CV” and “M” indicators. [file 12864_2020_7318_MOESM1_ESM.png]

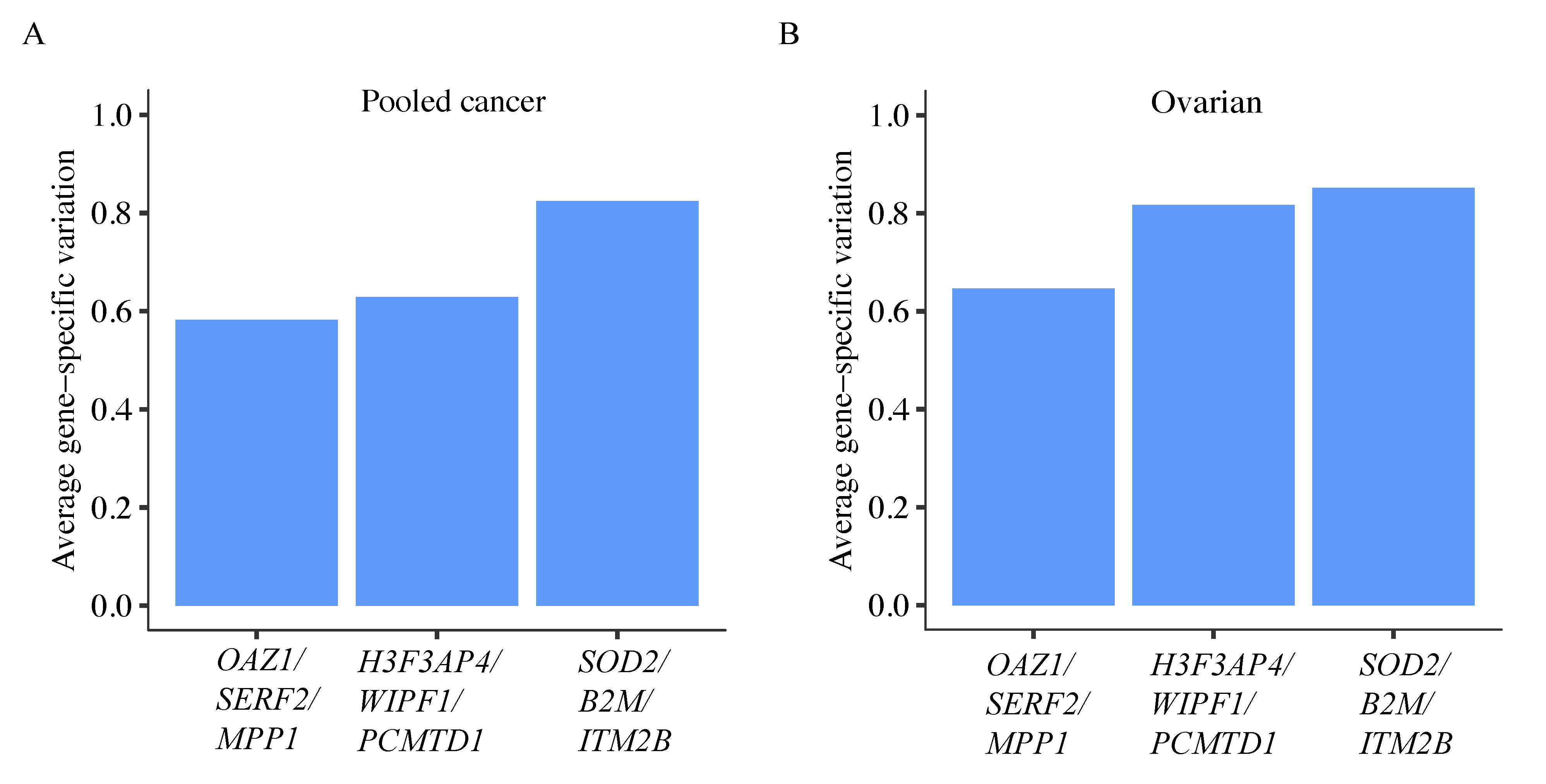

Supplement: Supplementary file 2 — Additional file 2: Figure S2. Evaluation of expression stability of combinations of three reference genes in pooled exosomes of cancer patients with PAAD, CRC and HCC (A, RNA-Seq data) and with ovarian cancer (B, qRT-PCR data) The expression stability of respective combinations was measured as the average gene-specific variation calculated with the geNorm algorithm based on transcripts per million (TPM) (A) or cycle threshold (Ct) values (B). Three combinations according to their expression stability ranking from Table 1 were evaluated: 1) genes 1–3 (OAZ1, SERF2, MPP1); 2) genes 4–6 (H3F3AP4, WIPF1, PCMTD1); and 3) genes 8–10 (SOD2, B2M, ITM2B) [file 12864_2020_7318_MOESM2_ESM.png]

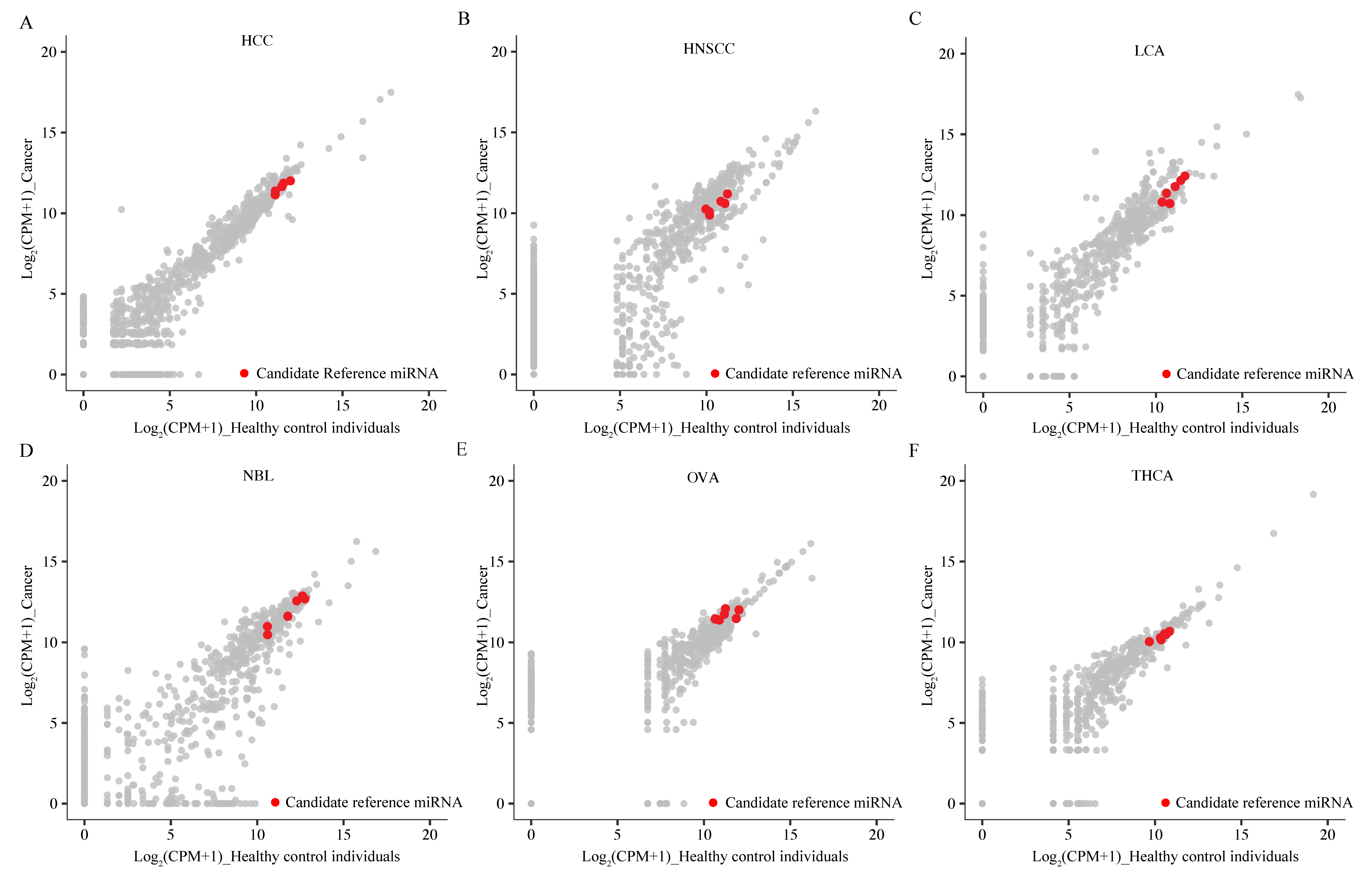

Supplement: Supplementary file 3 — Additional file 3: Figure S3. Scatterplots of expression levels of six candidate reference miRNAs (red dots) in serum exosomes of patients with HCC (A), HNSCC (B), LCA (C), NBL (D), OVA (E) and THCA (F) compared to exosomes of healthy control individuals The expression values are depicted as: log2(CPM + 1) (CPM – counts per million). Grey dots indicate genome-wide miRNAs [file 12864_2020_7318_MOESM3_ESM.png]

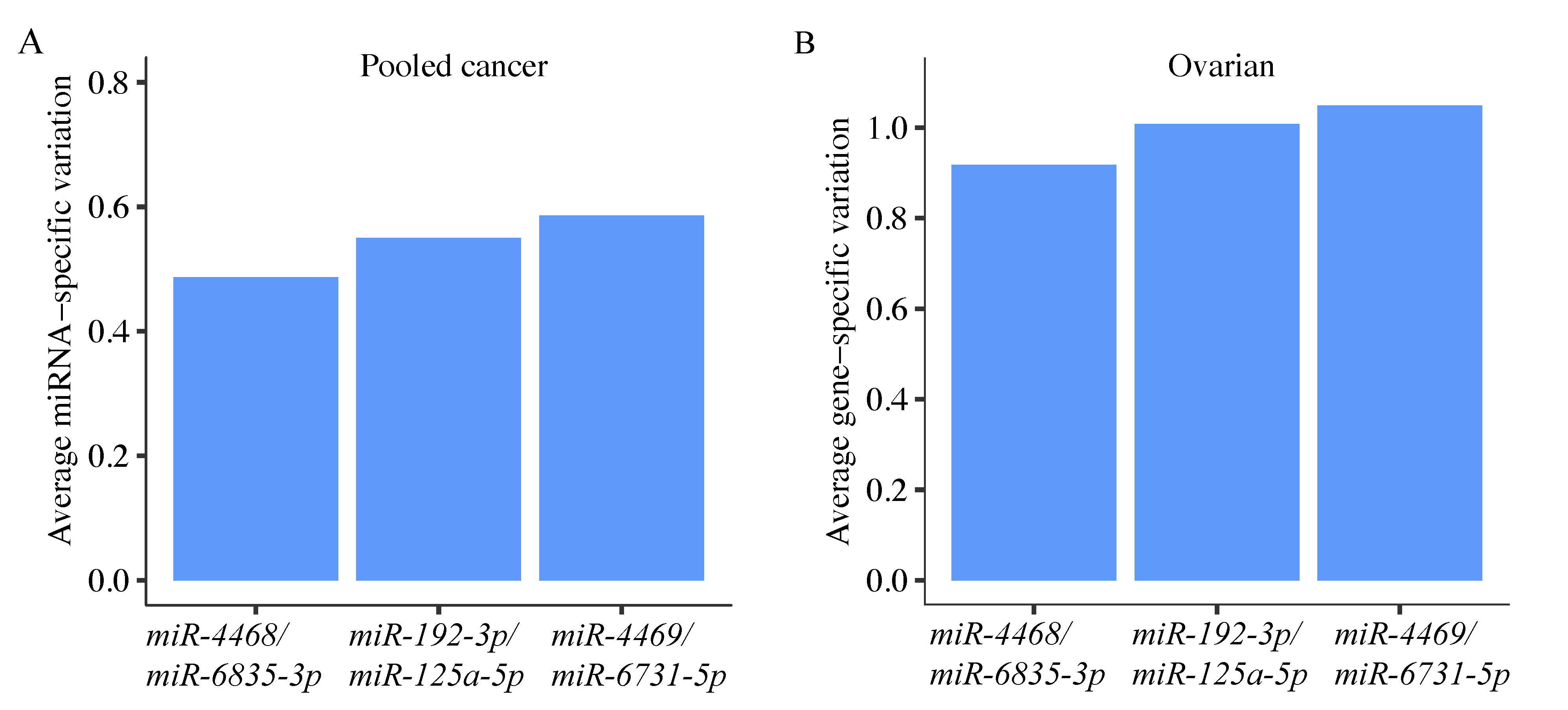

Supplement: Supplementary file 4 — Additional file 4: Figure S4. Evaluation of expression stability of combinations of two reference miRNA candidates in pooled exosomes of cancer patients with different tumor types (A, miRNA-Seq data) or with ovarian cancer (B, qPCR data) The expression stability of miRNA combinations was measured as the average miRNA-specific variation, which was calculated by the geNorm algorithm based on counts per million (CPM) (A) or cycle threshold (Ct) values (B). Three combinations were considered according to the miRNA expression stability ranks shown in Table 2: miRNAs 1–2 (miR-4468 and miR-6835-3p), miRNAs 3–4 (miR-192-3p and miR-125a-5p), and miRNAs 5–6 (miR-4469 and miR-6731-5p) [file 12864_2020_7318_MOESM4_ESM.png]

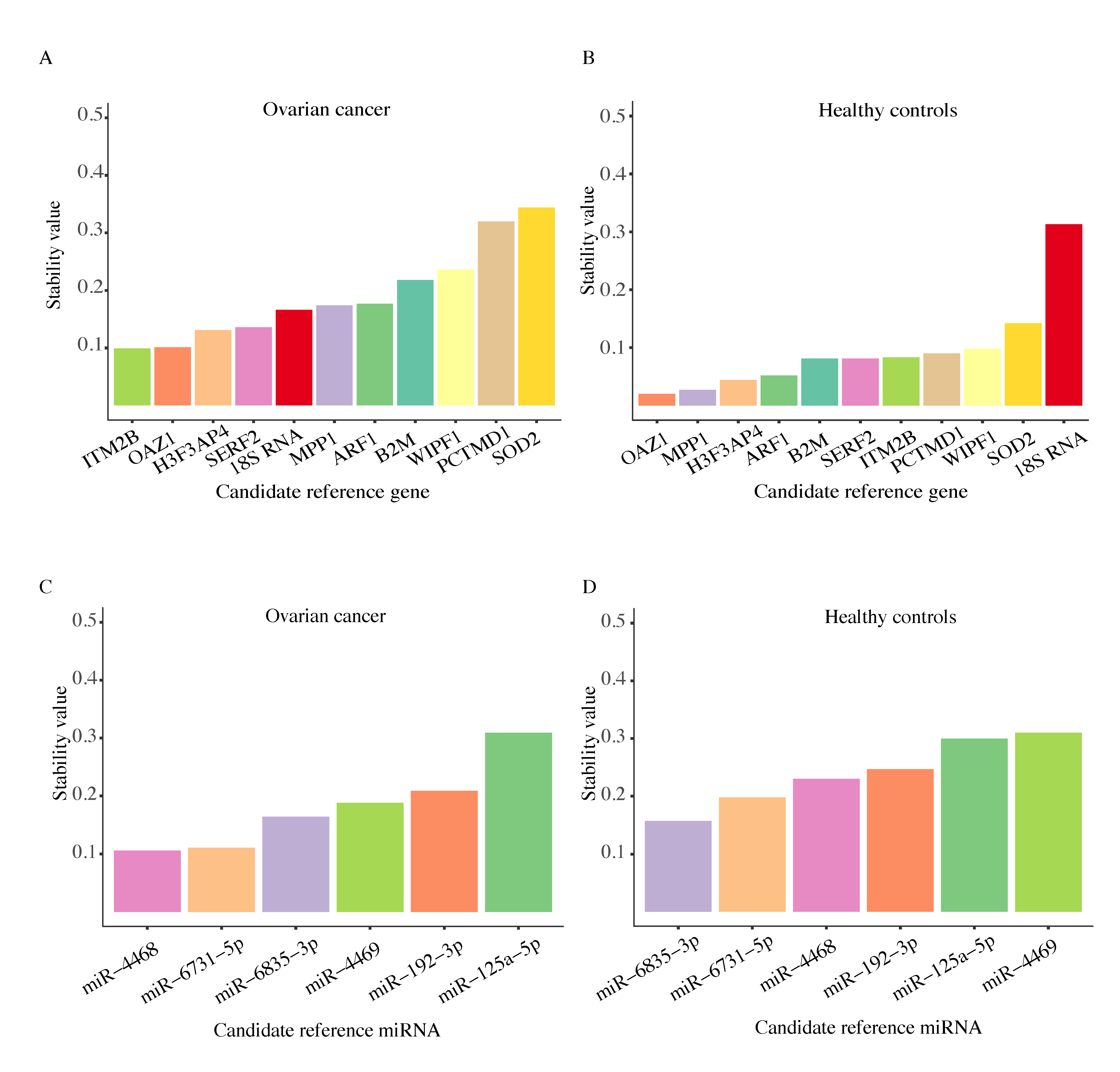

Supplement: Supplementary file 5 — Additional file 5: Figure S5. Validation of candidate reference genes and miRNAs predicted in exosomes of ovarian cancer patients and healthy controls by NormFinder Expression stability of candidate reference genes as measured by the NormFinder stability value in exosomes of ovarian cancer patients (A) and healthy control individuals (B). Expression stability of six candidate reference miRNAs in exosomes of ovarian cancer patients (C) and healthy control individuals (D) as measured by the NormFinder stability value [file 12864_2020_7318_MOESM5_ESM.png]

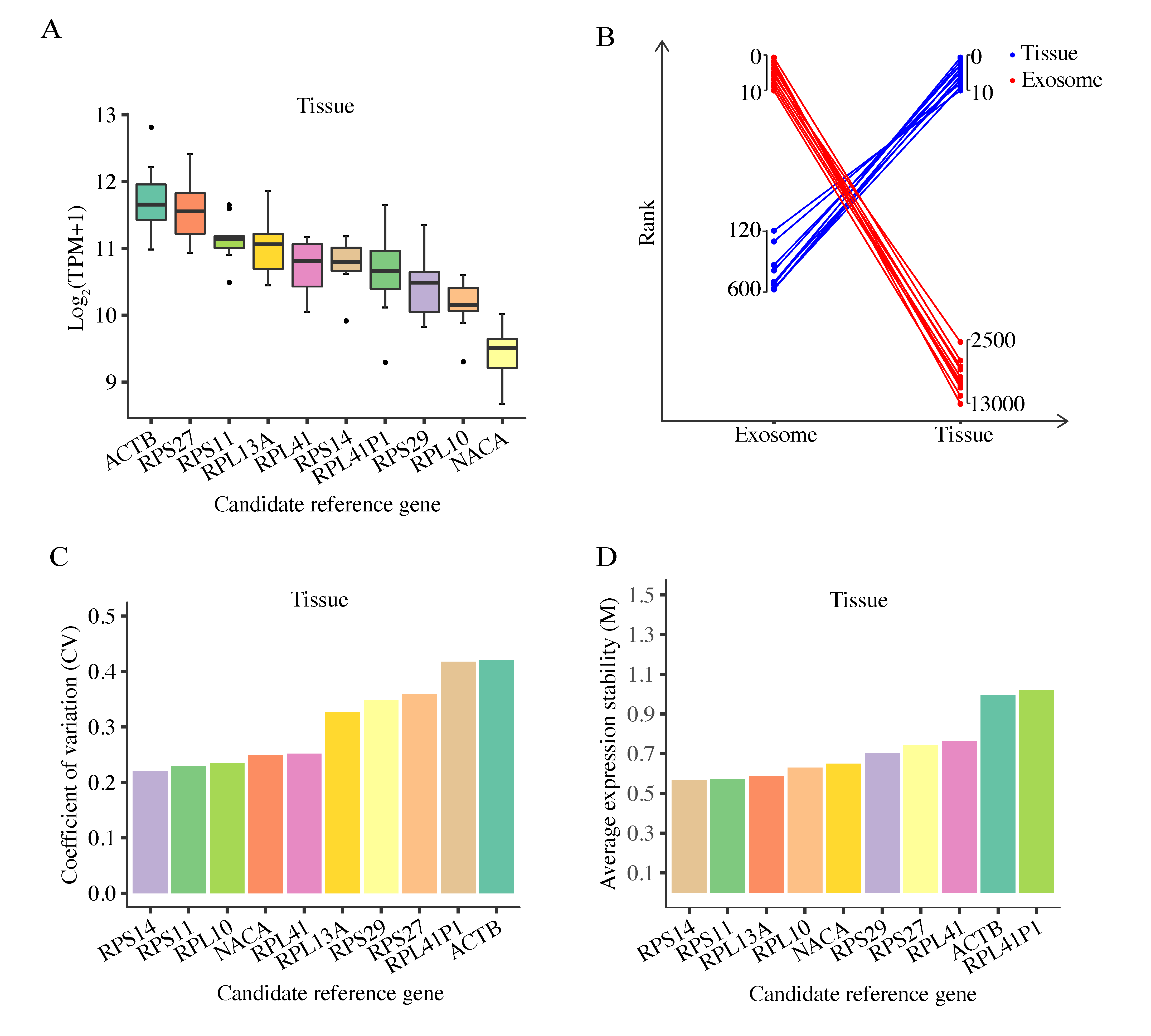

Supplement: Supplementary file 6 — Additional file 6: Figure S6. Analysis of candidate reference genes predicted in cancer tissues Expression levels of the ten top candidates in pooled cancer tissue samples calculated using RNA-Seq data. Expression levels are given as log2(TPM + 1; TPM = transcript per million) (A). The top ten predicted candidate reference genes for exosomes were compared with the respective ranking in cancer tissues and vice versa (B). Expression stability of the ten top candidate reference genes in tumor tissues as measured by “CV” (C) and “M” indicators (D) [file 12864_2020_7318_MOESM6_ESM.png]
