# Supplementary material for: Unbiased RNA-Seq-driven identification and validation of reference genes for quantitative RT-PCR analyses of pooled cancer exosomes
Source: BMC Genomics. 2021 Jan 6;22:27. doi: 10.1186/s12864-020-07318-y (PMC7789813; doi:10.1186/s12864-020-07318-y)
Supplement: Supplementary file 7 — Additional file 7: Table S1. List of candidate reference genes (n = 10) and miRNAs (n = 6) identified by RNA-Seq and quantitative real-time PCR analyses. [file 12864_2020_7318_MOESM7_ESM.docx]

**Table S1 List of candidate reference genes (n=10) and miRNAs (n=6) identified by RNA-Seq and quantitative real-time PCR analyses**

| **Type** | **Gene Symbol** | **Gene ID** | **Description** |
| --- | --- | --- | --- |
| mRNA | ARF1 | 375 | ADP ribosylation factor 1 |
| mRNA | B2M | 567 | beta-2-microglobulin |
| mRNA | H3F3AP4 | 440926 | H3 histone, family 3A, pseudogene 4 |
| mRNA | ITM2B | 9445 | integral membrane protein 2B |
| mRNA | MPP1 | 4354 | membrane palmitoylated protein 1 |
| mRNA | OAZ1 | 4946 | ornithine decarboxylase antizyme 1 |
| mRNA | PCMTD1 | 115294 | protein-L-isoaspartate (D-aspartate) O-methyltransferase domain containing 1 |
| mRNA | SOD2 | 6648 | superoxide dismutase 2 |
| mRNA | SERF2 | 10169 | small EDRK-rich factor 2 |
| mRNA | WIPF1 | 7456 | WAS/WASL interacting protein family member 1 |
| miRNA | hsa-miR-125a-5p | 406910 | microRNA 125a |
| miRNA | hsa-miR-192-3p | 406967 | microRNA 192 |
| miRNA | hsa-miR-4468 | 100616226 | microRNA 4468 |
| miRNA | hsa-miR-4469 | 100616115 | microRNA 4469 |
| miRNA | hsa-miR-6731-5p | 102465437 | microRNA 6731 |
| miRNA | hsa-miR-6835-3p | 102465502 | microRNA 6835 |
