# Supplementary material for: Unbiased RNA-Seq-driven identification and validation of reference genes for quantitative RT-PCR analyses of pooled cancer exosomes
Source: BMC Genomics. 2021 Jan 6;22:27. doi: 10.1186/s12864-020-07318-y (PMC7789813; doi:10.1186/s12864-020-07318-y)
Supplement: Supplementary file 8 — Additional file 8: Table S2. Detailed information for the RNA-Seq datasets in the GEO database. [file 12864_2020_7318_MOESM8_ESM.docx]

**Table S2 Detailed information for the RNA-Seq datasets in the GEO database**

| **GEO accession number** | **Type** | **Status** | **Samples** | **Bio Project** |
| --- | --- | --- | --- | --- |
| GSE100232 | exosome | pancreatic carcinoma | 14 | [PRJNA391134](https://www.ncbi.nlm.nih.gov/bioproject/PRJNA391134) |
| GSE100207 | exosome | hepatocellular carcinoma | 21 | [PRJNA390991](https://www.ncbi.nlm.nih.gov/bioproject/PRJNA390991) |
| GSE100206 | exosome | healthy | 32 | [PRJNA390988](https://www.ncbi.nlm.nih.gov/bioproject/PRJNA390988) |
| GSE100063 | exosome | colorectal carcinoma | 12 | [PRJNA390615](https://www.ncbi.nlm.nih.gov/bioproject/PRJNA390615) |
| GSE119794 | tissue | 10 paired tumour and normal pancreatic samples from patients | 40 | [PRJNA490335](https://www.ncbi.nlm.nih.gov/bioproject/PRJNA490335) |
| GSE79669 | cell line | pancreatic cancer | 14 | [PRJNA316672](https://www.ncbi.nlm.nih.gov/bioproject/PRJNA316672) |
| GSE79668 | tissue | 51 clinically annotated human pancreatic adenocarcinoma cancer tissue samples | 51 | [PRJNA316673](https://www.ncbi.nlm.nih.gov/bioproject/PRJNA316673) |
| GSE103383 | cell line | pancreatic beta cell line EndoC-BH1 | 27 | [PRJNA401709](https://www.ncbi.nlm.nih.gov/bioproject/PRJNA401709) |
| GSE84023 | cell line | pancreatic cancer | 73 | [PRJNA327834](https://www.ncbi.nlm.nih.gov/bioproject/PRJNA327834) |
| GSE96931 | cell line | pancreatic cancer | 15 | [PRJNA380131](https://www.ncbi.nlm.nih.gov/bioproject/PRJNA380131) |
| GSE94660 | tissue | 21 HBV-HCC patients with non-neoplastic liver and tumour tissue samples | 42 | [PRJNA371753](https://www.ncbi.nlm.nih.gov/bioproject/PRJNA371753) |
| GSE104310 | tissue | 10 tumours and paired non-tumour tissue samples | 20 | [PRJNA412314](https://www.ncbi.nlm.nih.gov/bioproject/PRJNA412314) |
| GSE119336 | tissue | 15 pairs of ICC tumour and matched non-tumour liver tissue samples | 30 | [PRJNA488803](https://www.ncbi.nlm.nih.gov/bioproject/PRJNA488803) |
| GSE83518 | cell line | hepatocellular carcinoma | 16 | [PRJNA326261](https://www.ncbi.nlm.nih.gov/bioproject/PRJNA326261) |
| GSE71446 | cell line | hepatocellular carcinoma | 12 | [PRJNA291214](https://www.ncbi.nlm.nih.gov/bioproject/PRJNA291214) |
| GSE93831 | cell line | hepatocellular carcinoma | 10 | [PRJNA362552](https://www.ncbi.nlm.nih.gov/bioproject/PRJNA362552) |
| GSE72820 | tissue | 7 matched colon adenoma and normal mucosa tissue samples | 14 | [PRJNA295149](https://www.ncbi.nlm.nih.gov/bioproject/PRJNA295149) |
| GSE86564 | tissue | paraffin-embedded colorectal cancer specimens | 54 | [PRJNA342194](https://www.ncbi.nlm.nih.gov/bioproject/PRJNA342194) |
| GSE50760 | tissue | normal colon, primary CRC, and liver metastasis | 54 | [PRJNA218851](https://www.ncbi.nlm.nih.gov/bioproject/PRJNA218851) |
| GSE109607 | cell line | colorectal cancer samples consisting of 6 samples for each of 5 cell lines | 30 | [PRJNA431467](https://www.ncbi.nlm.nih.gov/bioproject/PRJNA431467) |
| GSE75440 | cell line | colorectal cancer | 17 | [PRJNA304717](https://www.ncbi.nlm.nih.gov/bioproject/PRJNA304717) |
| GSE110114 | tissue | 10 samples of breast cancer tissue and 3 samples of adjacent normal tissue | 13 | [PRJNA432903](https://www.ncbi.nlm.nih.gov/bioproject/PRJNA432903) |
| GSE111073 | tissue | two samples from each of ten breast cancer patients | 21 | [PRJNA435751](https://www.ncbi.nlm.nih.gov/bioproject/PRJNA435751) |
| GSE104730 | tissue | male breast tumours | 46 | [PRJNA413693](https://www.ncbi.nlm.nih.gov/bioproject/PRJNA413693) |
| GSE71651 | tissue | 15 breast cancer and 18 normal tissue samples | 33 | [PRJNA292118](https://www.ncbi.nlm.nih.gov/bioproject/PRJNA292118) |
| GSE108541 | cell line | immortalized breast epithelial cell lines from normal breast tissue with a luminal or intrinsic subtype | 59 | [PRJNA427584](https://www.ncbi.nlm.nih.gov/bioproject/PRJNA427584) |
| GSE100075 | cell line | breast cancer cell line | 15 | [PRJNA390636](https://www.ncbi.nlm.nih.gov/bioproject/PRJNA390636) |
| GSE102616 | cell line | breast cancer cell line MCF7 | 47 | [PRJNA398222](https://www.ncbi.nlm.nih.gov/bioproject/PRJNA398222) |
| GSE81089 | tissue | fresh frozen tumour tissue samples from 199 patients diagnosed with NSCLC | 218 | [PRJNA320473](https://www.ncbi.nlm.nih.gov/bioproject/PRJNA320473) |
| GSE34914 | tissue | 8 lung adenocarcinoma with mutant KRAS and 8 lung adenocarcinoma without KRAS mutation tissue samples | 16 | [PRJNA150161](https://www.ncbi.nlm.nih.gov/bioproject/PRJNA150161) |
| GSE109720 | cell line | 4 different treated lung cancer cell lines | 33 | [PRJNA431798](https://www.ncbi.nlm.nih.gov/bioproject/PRJNA431798) |
| GSE123769 | cell line | lung adenocarcinoma | 18 | [PRJNA509770](https://www.ncbi.nlm.nih.gov/bioproject/PRJNA509770) |
| GSE111803 | exosome | 5 patients with lung adenocarcinoma and 5 healthy controls | 10 | [PRJNA438213](https://www.ncbi.nlm.nih.gov/bioproject/PRJNA438213) |
| GSE106277 | exosome | non-small cell lung cancer | 8 | [PRJNA416109](https://www.ncbi.nlm.nih.gov/bioproject/PRJNA416109) |
| GSE77318 | exosome | ovarian cancer | 13 | [PRJNA310092](https://www.ncbi.nlm.nih.gov/bioproject/PRJNA310092) |
| GSE128004 | exosome | neuroblastoma patients and normal controls | 18 | [PRJNA526037](https://www.ncbi.nlm.nih.gov/bioproject/PRJNA526037) |
| GSE84306 | exosome | 4 distinct head and neck squamous cell carcinoma (HNSCC) cell lines and normal oral epithelial cells | 20 | [PRJNA328779](https://www.ncbi.nlm.nih.gov/bioproject/PRJNA328779) |
| GSE130512 | exosome | 8 benign thyroid nodules and 16 PTC patients with or without metastasis | 24 | [PRJNA540503](https://www.ncbi.nlm.nih.gov/bioproject/PRJNA540503) |
| GSE104251 | exosome | 5 hepatocellular carcinoma and 5 liver cirrhosis tissue samples | 10 | [PRJNA412166](https://www.ncbi.nlm.nih.gov/bioproject/PRJNA412166) |
